# City-scale Vehicle Trajectory Data from Traffic Camera Videos

**DOI:** 10.1038/s41597-023-02589-y

**Published:** 2023-10-17

**Authors:** Fudan Yu, Huan Yan, Rui Chen, Guozhen Zhang, Yu Liu, Meng Chen, Yong Li

**Affiliations:** 1 Beijing National Research Center for Information Science and Technology (BNRist), Beijing, 100084 China; 2 Department of Electronic Engineering, Tsinghua University, Beijing, 100084 China; 3 School of Software, Shandong University, Jinan, 250101 China

**Keywords:** Social sciences, Research data

## Abstract

Vehicle trajectory data underpins various applications in intelligent transportation systems, such as traffic surveillance, traffic prediction, and traffic control. Traditional vehicle trajectory datasets, recorded by GPS devices or single cameras, are often biased towards specific vehicles (e.g., taxis) or incomplete (typically < 1 km), limiting their reliability for downstream applications. With the widespread deployment of traffic cameras across the city road network, we have the opportunity to capture all vehicles passing by. By collecting city-scale traffic camera video data, we apply a trajectory recovery framework that identifies vehicles across all cameras and reconstructs their paths in between. Leveraging this approach, we are the first to release a comprehensive vehicle trajectory dataset that covers almost full-amount of city vehicle trajectories, with approximately 5 million trajectories recovered from over 3000 traffic cameras in two metropolises. To assess the quality and quantity of this dataset, we evaluate the recovery methods, visualize specific cases, and compare the results with external road speed and flow statistics. The results demonstrate the consistency and reliability of the released trajectories. This dataset holds great promise for research in areas such as unveiling traffic dynamics, traffic network resilience assessment, and traffic network planning.

## Background & Summary

Vehicle trajectory data records the movement paths of vehicles, including their position and corresponding timestamp. It has been widely applied in transportation applications such as traffic surveillance^1,2^, traffic prediction^3–5^, and traffic management^6–8^. In the context of traffic surveillance, this data enables the identification of congested areas, traffic hotspots, and accident-prone zones. In traffic prediction, it is leveraged to forecast future traffic conditions using historical trends and predictive models. Moreover, in traffic management, vehicle trajectory data plays a critical role in optimizing traffic flow and identifying bottlenecks for effective traffic management strategies. Existing vehicle trajectory datasets can be divided into two categories by data source: GPS-based datasets and video-based datasets. The GPS-based vehicle trajectory data can be collected in large amounts, and cover a wide spatial area with the popularity and capacity of GPS devices. However, these datasets often have biases towards certain types of vehicles, such as buses^1^ and taxis^9–12^, or specific groups of drivers, such as navigation users^13^ or ride hailing drivers^14^, due to privacy concerns. On the other hand, traditional video-based datasets^15–20^ track and detect vehicles within the field of view of a single camera. Although high-resolution cameras with wide fields of view, such as high-flying drones^15–20^, are used, the recorded trajectories are still limited in length (typically less than 1 km^16–18,20^), which prevents the recording of all-amount trajectories.

With the widespread deployment of traffic cameras in modern cities, there is a growing opportunity to capture complete trajectories of vehicles. Traffic camera videos faithfully record all passing vehicles, offering a promising and unbiased source of vehicle trajectories. With sufficient camera coverage, these videos have the potential to capture nearly full-amount of vehicle trajectories. By utilizing vehicle re-identification techniques across different cameras in the city’s traffic camera system^21,22^, it becomes possible to reconstruct the complete trips of vehicles on the road network. The existing datasets^23,24^ generated from traffic cameras are synthetic vehicle trajectories derived from either aggregated traffic information or trajectory distribution. The dataset from Wang *et al*.^23^ focuses on aggregated flow-speed data and utilizes resampling techniques to generate holographic trajectories. In contrast, the dataset released by Li *et al*.^24^ generates synthetic individual-level trip data based on statistical frequency, providing information such as origin, departure time, destination, and path. However, it’s important to note that both datasets assume near-complete coverage of traffic cameras across all road intersections and the flawless operation of optical character recognition (OCR) based license plate recognition systems. In reality, these conditions are often not met in most cities today^25,26^, which presents challenges for vehicle re-identification and trajectory recovery that may exceed the claims made in these studies.

**Fig. 1:**
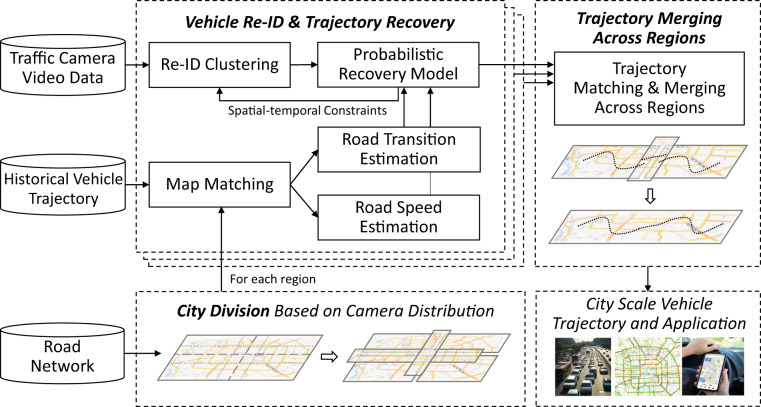
Overall framework of the city-scale vehicle trajectory recovery system.

In this paper, to the best of our knowledge, we are the first to release a city-scale traffic-camera-based vehicle trajectory dataset. The dataset contains almost full-amount of vehicle trajectories, comprising approximately 5 million trajectories obtained from four days of camera videos collected from over 3000 traffic cameras in two Chinese metropolises. In order to recover the complete trip of each vehicle from the raw video data, we employ a trajectory recovery framework. As shown in Fig. 1, the core of the framework is an iterative spatial-temporal vehicle re-identification (Re-ID) and trajectory recovery system. This system optimizes both the Re-ID task and the path inference task by incorporating valuable spatial-temporal knowledge extracted from historical GPS-based vehicle trajectories. To address computational constraints, we partition the city road network into regions based on the distribution of traffic cameras. The trajectory recovery system is applied to each region, and then a trajectory merging operation is performed across regions to connect trajectories that were split apart during the division process. To show the spatial-temporal characteristics of the dataset, we visualize the data distribution in terms of static attributes and dynamic flow. We also evaluate the quality and quantity of the dataset by assessing the performance of our recovery method, visualizing specific resulting cases, and comparing road speed and flow with external statistical data.

This comprehensive dataset of individual-level vehicle trajectories at a city-scale level holds great potential for various research directions and downstream applications. Its availability of unbiased, full-amount data is expected to yield compelling research findings and more robust management strategies. Here are some potential tasks that this dataset can support:

- **Unveiling city-scale traffic dynamics**. By leveraging city-scale full-amount trajectory data and employing advanced data-driven methods, we can uncover valuable insights into traffic patterns and the intricate relationships among different traffic variables, unveiling the dynamics of traffic in the city^27^. This understanding is essential for gaining a deeper comprehension of the underlying mechanisms that drive urban transportation operations, as well as understanding the impact of urban traffic on the environment^28^, allowing us to develop effective traffic management strategies to enhance overall system efficiency in cities.
- **Traffic network resilience assessment**. By combining knowledge of traffic demand, road segment speeds, and road network topology, it becomes possible to identify the critical bottlenecks and assess the overall resilience of the urban road network^29^. This understanding enables proactive measures to address vulnerabilities and enhance accessibility, playing a pivotal role in promoting smooth and efficient mobility for individuals and communities within the city.
- **Traffic network planning**. The dataset, with its comprehensive traffic demand and flow distribution records, can aid in identifying hotspots and congested roads in urban areas. This enables data-driven approaches for optimizing traffic network planning, encompassing the enhancement of road infrastructure, optimization of public transportation routes, and strategic placement of Point of Interest (PoI) locations^30–32^. Effective traffic network planning plays an important role in reducing traffic congestion and optimizing traffic flow, leading to a more efficient and sustainable urban environment.

## Methods

**Fig. 2:**
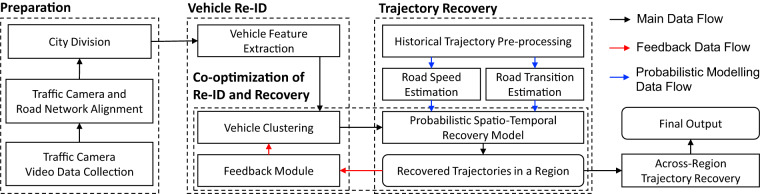
Data flow of our approach.

Our framework, as depicted in Fig. 2, follows a streamlined data flow. Initially, we collect traffic camera video data from over 3000 cameras in two Chinese metropolises. The raw video data is then sampled and cropped into images. These images undergo feature extraction, generating visual features. These features are subsequently inputted into a clustering module, which identifies clusters representing vehicles re-identified across multiple cameras. Next, a probabilistic trajectory recovery model utilizes these clusters to infer the travel path between camera observations. To improve accuracy, a feedback data flow enables co-optimization of the re-identification and recovery processes. Additionally, spatial-temporal information from historical trajectories supports the probabilistic model through a dedicated data flow. Finally, within the divided city regions, an across-region trajectory recovery module is employed to match and merge trajectories that may have been split during the city division process.

### Ethics statement

In this section, we state how all the input data in this study are properly processed and used so that no personally identifying information is disclosed in any form. We further describe how the non-public input data is used under the awareness and approval of third-party data providers, ensuring that both the study and the released dataset strictly adhere to the stipulated collaboration terms. There are three kinds of input data in this study, including traffic camera video data, road network data, and historical trajectory data. The traffic camera videos utilized in this study are non-public data, acquired through collaborative efforts with third-party agencies. To protect the privacy of individuals featured in the videos, third parties anonymized the videos by only providing visual feature vectors embedded by a confidential neural network so that it is impossible to infer the real-world vehicle plate ID. Our methodology enables the differentiation of vehicles in videos by measuring the distance between these vectors, not by attempting to infer the real-world vehicle plate IDs. Consequently, the proposed trajectory dataset does not contain any real-world vehicle identity information. Further, we are under the awareness and approval of the third parties to release the proposed trajectory dataset where we do not publish the anonymized vectors in any form, while the spatial-temporal information of trajectory points is partly inferred based on the camera position and camera shooting time. For road network data, we obtain, use and re-distribute this public data under the Open Data Commons Open Database License 1.0 adopted by OpenStreetMap. The historical trajectory data, obtained through collaboration with Amap, a major location-based service provider in China, is non-public. The data is collected by Amap with users’ consent during the provision of map routing services through their app. To protect the privacy of the users, Amap removed the “UserID” attribute before providing us the data, ensuring no personally identifying information could be disclosed. We have the awareness and approval of Amap to release the proposed trajectory dataset. It includes only the trajectories recovered from traffic camera videos on the respective days of collection; no anonymized historical trajectories are published in any form. It should be emphasized that, while the dataset may reflect coarse-grained statistical characteristics analogous to the historical anonymized trajectory data, there is no release of the actual anonymized data.

### Traffic camera video data collection

We collected raw traffic camera video data from two Chinese metropolises: Shenzhen and Jinan. Shenzhen is renowned as one of China’s special economic zones and a global financial center, while Jinan serves as the capital of Shandong province in Eastern China. In Shenzhen, we collected videos from three specific days: November 4, 2020 (Wednesday), April 16, 2021 (Friday), and August 24, 2021 (Tuesday). These videos were captured from a varying number of cameras on each day. Specifically, on November 4, 2020, we collected videos from 441 cameras. These cameras covered a major city district in Shenzhen, known as the Longhua district. On April 16, 2021, we expanded our coverage to four city districts: Longhua, Guangming, Nanshan, and Futian. Hence, we collected videos from 1,460 cameras on that day. Similarly, on August 24, 2021, videos were collected from the same set of 1,460 cameras in these four city districts. The data collection starts from 8 a.m. to 8 p.m. In Jinan, we collected videos for a single day: October 17, 2022 (Monday). The videos were obtained from a total of 1,838 cameras, providing comprehensive coverage of the entire city of Jinan. The data collection spanned the entire day, starting from midnight (0 a.m.) and continuing until midnight (24 p.m.).

The video data in Shenzhen city was obtained through a collaboration with SenseTime (https://www.sensetime.com/), a company specializing in AI technologies for various domains such as smart city. Similarly, the traffic camera video data in Jinan city was provided by the Jinan traffic management agency (https://jnjtj.jinan.gov.cn/) through a collaboration with the authors. Please note that the video data used in this study is not publicly available. Researchers interested in replicating or conducting similar studies can explore collaboration opportunities with traffic agencies or companies specializing in traffic surveillance technologies and devices.

To process the raw video data, we employ a sampling technique where frames are extracted from the videos at a regular interval of 2 seconds. Each frame is then converted into an image. Subsequently, a state-of-the-art, pre-trained Convolutional Neural Network (CNN)-based vehicle detection model is utilized to identify and isolate vehicles within each frame image. These detected vehicles are cropped into smaller images, which we refer to as “camera records” for individual vehicles. As a result of this process, we obtained a substantial number of camera records from the collected videos. Specifically, in Shenzhen, approximately 4 million, 13 million, and 14 million camera records were extracted from the videos recorded on November 4, 2020, April 16, 2021, and August 24, 2021, respectively. In Jinan, around 19 million camera records were extracted from the videos recorded on October 17, 2022. These camera records serve as the foundation for our dataset and provide detailed information about each detected vehicle captured by the traffic cameras. The statistics of the collected data are summarized in Table 1.

**Table 1:** Spatial-temporal ranges and quantities of the dataset.

City	Date	Spatial Range	Temporal Range	#Camera	#Camera Record
Shenzhen	2020.11.04	1 district	8 a.m. - 8 p.m.	441	4 million
	2021.04.16	4 districts		1460	13 million
	2021.08.24				14 million
Jinan	2022.10.17	whole city	all day long	1838	19 million

Finally, to properly protect the privacy of all the citizens, who may be unaware their driving trajectories are shared (though they know that these are captured by traffic cameras), both SenseTime and the Jinan traffic management agency anonymized the video data in line with ethical procedures before providing it to us. Specifically, the raw camera records with potential personal identifiers are transformed into 256-dimensional vectors using a CNN before reaching us. The trained CNN’s model parameters remain confidential, ensuring that no personal details like vehicle license plates can be deciphered from these vectors. Besides, our released dataset only includes trajectories without real-world identity information, excluding these vectors.

### Traffic camera and road network alignment

**Fig. 3:**
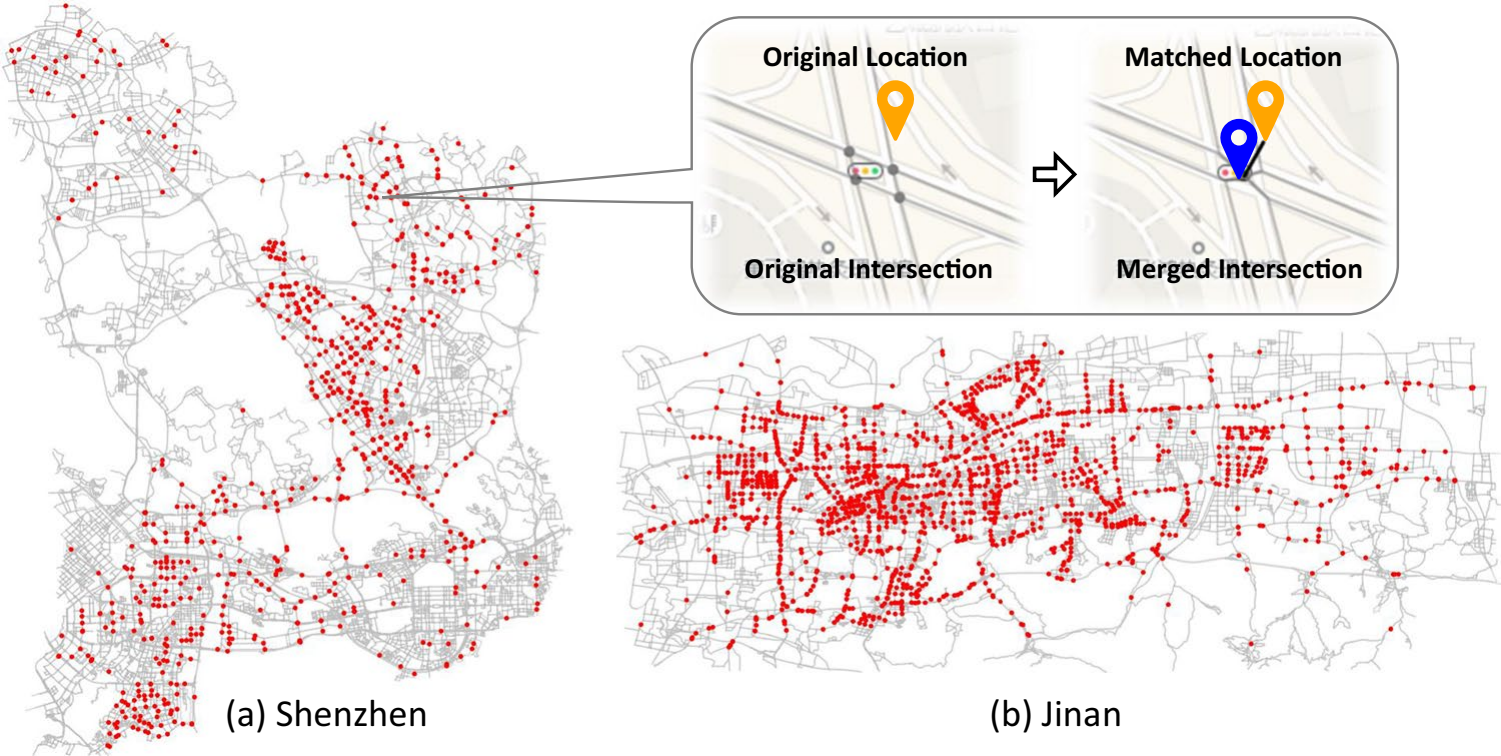
Spatial distribution of traffic cameras in the road network.

We acquired road network data from OpenStreetMap^33^, a freely available geographic database maintained by a community of volunteers. Researchers interested in replicating or conducting similar studies can access road network data directly from OpenStreetMap. We build a directed graph *G* = <*V*, *E*> to represent the road network, where *V* and *E* are road intersections and roads respectively, with the Python package OSMnx^34^. The coordinates of traffic cameras are mainly provided by government, and some missing cases are complemented by resorting to Geocoding^35^. Then we align traffic cameras to *G* by matching each camera to a graph node. In order to ensure that one logical road intersection is exactly represented by one single node in *G*, any group of neighboring nodes is detected with a spatial threshold *D* and merged into a single node. Then, we match each traffic camera to the nearest graph node within a spatial threshold *D*. However, while most traffic cameras are deployed at road intersections and thus can be matched to a graph node, some cameras are deployed in the middle of roads and there are no nearby nodes. In this case, we project a camera to its nearby roads. Note that we project the camera to all nearby roads as long as the corresponding projection point is within a threshold *D*, rather than only project the camera to the nearest road, considering that cameras typically monitor all the parallel neighboring roads in opposite directions. Then we introduce a new node at the center of all the projection points, cut apart and re-link the projected roads through the new node. In the end, we obtain a new graph *G*′ = <*V*′, *E*′> with some nodes merged into a single node, some new nodes introduced as projection points, and some roads divided apart at projection points. In this way, each traffic camera can be matched to its nearest node. In Shenzhen city, 1365 out of 1460 cameras are matched to 686 nodes within distance threshold *D*, where some cameras are not matched due to incompleteness of road network and possible coordinate errors of traffic cameras, and multiple cameras can be matched to the same node since several cameras can be deployed at one intersection. Since it is observed that 94% of cameras are located within a distance of 20 m from the road network, we set the threshold D to 20 m. Figure 3 shows the cameras and road networks in Shenzhen and Jinan. Additionally, a special case is presented to illustrate how 4 original graph nodes are merged together to represent a road intersection, and how a camera is matched to the road intersection.

### City division

Due to computational constraints, it is not feasible to perform vehicle re-identification and trajectory recovery over the entire city with thousands of traffic cameras and millions of camera records. To address this issue, we propose dividing the city area into several regions, each containing hundreds of cameras and millions of records, making it feasible for our algorithms. However, this division may result in incomplete trajectories for vehicles crossing region boundaries. We alleviate this by considering three aspects: (1) Each region is kept sufficiently large to accommodate the majority of vehicles traveling within it. (2) The region boundaries are determined by clustering the distribution of traffic cameras, resulting in sparser camera density at the boundaries and minimizing information loss during the division. (3) We design a trajectory matching and merging algorithm to connect and combine trajectories across regions (see following section “Across-Region Trajectory Recovery”).

Specifically, we utilize a size constrained clustering approach^36^ based on the coordinates of traffic cameras. This method extends the original K-Means algorithm by imposing constraints on the minimum and maximum size of each cluster. This ensures that each region has a minimum number of cameras to maintain its spatial scale and a maximum number of cameras to ensure computational feasibility. We conduct the experiments with different numbers of clusters to generate various city division plans and evaluate their quality using the Silhouette Coefficient, which measures the compactness and separation of clusters. Based on this evaluation, we select the best city division plan. After obtaining the regions through clustering, we introduce additional *auxiliary regions* along the boundaries between the divided regions. These auxiliary regions play a crucial role in matching and merging trajectories across the divided regions, facilitating a more complete reconstruction of vehicle trajectories.

### Vehicle Re-identification (Re-ID)

In vehicle Re-ID, the sampled and cropped images extracted from video data are initially transformed into visual features. These visual features are then input into a clustering module, which is used to generate clusters representing vehicles that have been re-identified at multiple cameras.

#### Vehicle feature extraction

Towards vehicle Re-ID, we apply modern pretrained CNN-based methods to extract vehicle visual features^21^. Given a camera record, namely, a small image of one detected vehicle cropped from a video frame, we use a ResNet-50 backboned model to extract a general appearance feature *f*_*a*_. We further apply a license plate detection model to crop vehicle image into license plate image and use a ResNet-50 backboned model to extract a license plate feature *f*_*p*_. For some vehicles *f*_*p*_ is not available because the license plate detection can fail when vehicle image is of poor quality, for example, poor resolution, bad lighting, skewed or blocked sight line. *f*_*a*_ and *f*_*p*_ as the output of neural network model are 256-dimensional vectors. For efficiency of the following processes, we apply the principal components analysis (PCA) in each city region to reduce their dimension to 64. Besides, in order to incorporate spatial-temporal information into the re-identification task, we also introduce a dynamic feature *f*_*d*_ which is initialized the same as *f*_*a*_ and updated by our following spatial-temporal models (see following section “Co-optimization of Vehicle Re-ID and Trajectory Recovery”).

Note that we do not employ the license plate recognition (LPR) approach suggested by the work^23^ for two reasons. Firstly, license plate detection can be unreliable due to various factors such as camera resolution, lighting conditions, and obstructions, resulting in a significant number of records without LPR results. Secondly, LPR is prone to errors when dealing with poor-quality images, often leading to incorrect identification of characters such as “0” and “D”. In fact, a related work^26^ conducts a study on how LPR performance deteriorates when provided with traffic camera snapshots instead of high-quality images as in an automatic parking payment system, where less than 10% of the snapshots are completely correctly recognized by LPR.

#### Vehicle clustering

A 2-stage vehicle clustering algorithm is designed based on the extracted vehicle features. The first stage searches the top *k* nearest neighbors for each vehicle record based on its appearance feature *f*_*a*_ and plate feature *f*_*p*_ respectively. Considering the quadratic complexity, we implement this by Faiss^37^, which is a library for efficient similarity search of dense vectors. The second stage is a multi-modal similarity clustering process with linear complexity. The similarity between two records *i* and *j* is defined as the weighted average of cosine similarities based on appearance feature *f*_*a*_, plate feature *f*_*p*_ and dynamic feature *f*_*d*_ (all 256-dimensional vectors), with corresponding scalar weights *w*_*a*_, *w*_*p*_ and *w*_*d*_.

1 $$Si{m}_{i,j}=\left(\begin{array}{cc}\frac{{w}_{a}\,{f}_{a}^{i}\cdot {f}_{a}^{j}+{w}_{p}\,{f}_{p}^{i}\cdot {f}_{p}^{j}+{w}_{d}\,{f}_{d}^{i}\cdot {f}_{d}^{j}}{{w}_{a}+{w}_{p}+{w}_{d}}, & {\rm{if}}\,{f}_{p}^{i}\;{\rm{and}}\,{f}_{p}^{j}\;{\rm{available,}}\\ \frac{{w}_{a}{f}_{a}^{i}\cdot {f}_{a}^{j}+{w}_{d}\,{f}_{d}^{i}\cdot {f}_{d}^{j}}{{w}_{a}+{w}_{d}}, & {\rm{if}}\,{f}_{p}^{i}\;{\rm{or}}\,{f}_{p}^{j}\;{\rm{unavailable}}{\rm{.}}\end{array}\right.$$

The similarity between a record and a cluster is defined as the average similarity between the record and each record in cluster. For each record, we define its *candidate records* as the searched KNN records, and its *candidate clusters* as the clusters that *candidate records* belong to. Then clusters are progressively constructed by traversing the records once. For a current target record during traversing, we find the cluster with the maximum similarity among its *candidate clusters*. Then the record is added to this cluster if the maximum similarity is larger than a threshold *S*, else a new cluster is built for this record.

The parameter settings are as follows. We set *k* to search KNN records is 128, with similarity weights *w*_*p*_ = 0.8, and *w*_*a*_ = *w*_*p*_ = 0.1. Additionally, the similarity threshold is set to *S* = 0.8, as suggested in our previous works^21,22^. First, we mainly design the first stage KNN search to reduce the search scope in the second stage, which is of higher computational cost than the first stage. While our intention is to achieve results similar to an infinite *k* setting, we gradually reduce the value of *k* from a large number and observe how the final cluster results change. As a result, we find a value of 128 for *k* is sufficiently large to ensure that the final neighbors are included in the KNN candidates. Then, for the allocation of similarity weights to different visual features, our insight is that a larger weight should be assigned to the license plate feature *f*_*p*_ compared to the general appearance feature *f*_*a*_. This is because different vehicles can exhibit similar appearances, but the same vehicle can appear significantly different under varying lighting conditions. Next, we perform a grid search to determine the specific ratio between *w*_*p*_ and *w*_*a*_ while keeping *w*_*d*_ = *w*_*a*_, given that *f*_*d*_ is initialized the same as *f*_*a*_. Finally, the similarity threshold *S* is also decided through a grid search. Further details can be found in our prior work^22^.

### Vehicle trajectory recovery

Camera records are grouped into clusters using vehicle Re-ID, which yield multiple spatial-temporal observations of a vehicle at different road intersections. However, it is not sufficient to accurately recover the complete trajectory due to the possibility of multiple paths between consecutive observations. To overcome this challenge, we incorporate historical vehicle trajectories and employ a probabilistic model^22^ that leverages spatial-temporal information from historical vehicle trajectories. This model helps to infer the most possible path among the uncertain alternatives, enhancing the trajectory recovery process.

#### Probabilistic spatial-temporal recovery model

We adopt the probabilistic spatial-temporal recovery model from our previous work^22^. It can calculate the probability of any path between two consecutive camera observations given the knowledge extracted from historical trajectories, thus helps find the most probable path. Comparing with neural network based models, the probabilistic model is highly explainable and, more importantly, efficient, and thus applicable in city-scale scenarios.

Specifically, given two chronologically consecutive camera observations, i.e., the start record point *r*_*s*_, start time *t*_*s*_, end record point *r*_*e*_ and end time *t*_*e*_ = *t*_*s*_ + Δ*t*, we denote the trajectory connecting the two points as *P* = {*s*_1_, …, *s*_*n*_}, where *s*_*i*_ represents the road segment. The posterior probability of the trajectory given the above information can be factorized into two parts:

2 $$\Pr (p| {r}_{s},{t}_{s},{r}_{e},{t}_{e})\propto \Pr (p,\Delta t| {r}_{s},{r}_{e},{t}_{e})=\Pr (p| {r}_{s},{r}_{e},{t}_{e})\Pr (\Delta t| p,{t}_{e}).$$

The first factor is a prior probability that drivers who intend to move from *r*_*s*_ to *r*_*e*_ will choose this trajectory as their route around time *t*_*e*_, which accounts for the general popularity of the trajectory. The second factor is the likelihood that Δ*t* is taken to travel along this trajectory around time *t*_*e*_, which accounts for the consistency between the actual travel time and the expected travel time determined by the real-time traffic condition. To consider the effect of *t*_*e*_, practically we quantize it into 24 time slots with one-hour resolution, and the spatial-temporal knowledge extracted from historical trajectories in corresponding time slot will be used.

To model the prior factor, we assume that the transition from one road segment to another is independent from the start point *r*_*s*_ given the end point *r*_*e*_ and satisfies Markov property:

3 $$\Pr \left(p| {r}_{s},{r}_{e},{t}_{e}\right)=\Pr \left({s}_{1},\ldots ,{s}_{n}| {r}_{s},{r}_{e},{t}_{e}\right)=\Pr \left({s}_{1}| {r}_{s},{r}_{e},{t}_{e}\right)\mathop{\prod }\limits_{i=1}^{n-1}\Pr \left({s}_{i+1}| {s}_{i},{r}_{e},{t}_{e}\right).$$

We refer to $\Pr ({s}_{1}| {r}_{s},{r}_{e},{t}_{e})$ as start segment probability and $\Pr (s{\prime} | s,{r}_{e},{t}_{e})$ as segment transition probability. The start segment probability is calculated as the sum of segment transition probability over all the incoming segments.

To model the likelihood factor, we assume that the relative deviation of average speed follows a normal distribution:

4 $$\Pr \left(\Delta t| p,{t}_{e}\right)=\exp \left(-{\left(\overline{\Delta t}/\Delta t-1\right)}^{2}/2{\sigma }^{2}\right),$$

where $\overline{\Delta t}$ is the sum of the average traveling time of each segment in *p* at time slot *t*_*e*_, and *σ* is a hyper-parameter fine-tuned around the statistical standard deviation of historical trajectories.

#### Historical trajectory pre-processing

We collected historical GPS-based vehicle trajectories from two cities, provided by Amap (https://mobile.amap.com/), a prominent map service provider in China through our collaboration. However, it should be noted that this specific dataset is not publicly available. Researchers interested in similar data can explore collaboration opportunities with navigation service providers, ride-hailing platforms, taxi operators, or government agencies. To state the ethics of using this data and avoid privacy issues, the Amap users have given consent for the APP to collect their trajectories for research to improve location-based services. Further, Amap deleted the “UserID” attribute before providing us with the data, ensuring only anonymized trajectories are available. What’s more, we use this data only to estimate road speeds and transition patterns for our trajectory recovery model and do not share these historical trajectories in any form.

In Shenzhen, there are 2,111,309 trajectories of 824,929 Amap users within 2 weeks. In Jinan, there are 1,941,272 trajectories of 716,337 Amap users within 1 month. Although the historical trajectories may not encompass the full-amount vehicles, the dataset benefits from weeks of data collected from nearly one million navigation APP users. As a result, it is anticipated that the dataset will encompass a substantial number of samples representing various types of vehicles. This wealth of diverse data provides a solid foundation for robustly estimating average road speed and road transition patterns.

To align the trajectories with the road map, we utilize a modern and efficient map-matching algorithm^38^. This algorithm is specifically designed to handle large volumes of GPS points and road edges, making it suitable for our city-scale scenario. We carefully analyze the map-matched results and find that due to some complex city road network structures, such as complex intersections with rotary interchanges and overpasses, the map-matched paths can generate many unreal rings when some GPS points shift to a nearby road due to GPS error. When the nearby road is however quite distant at the topology view, a ring with multi-hops is generated to travel to-and-fro. We design a heuristic treatment which performs appropriate down-sampling to the input GPS points near complex road structures so that less GPS errors can occur in this critical regions. Meanwhile, it removes the rings in the results when few GPS points are map-matched on the ring.

#### Road speed estimation

To model the likelihood factor (Eq. 4), we estimate the vehicle speeds on each road in each time slot (one hour) based on the map-matched historical trajectories. However, we find it non-trivial because the distribution of historical trajectories on different roads is unbalanced and some roads suffer from sparsity. And since the speed is estimated in each time slot, the sparsity issue can deteriorate. Also, due to the restricted sampling rate of GPS points and large vehicle speeds, it is not rare that only one or even no GPS point is recorded at one road among a vehicle’s trajectory, and in this case it is not straightforward to calculate the speed of that road.

To tackle these problems, we first calculate road speeds with a set of algorithms designed for different situations for a road depends on how many GPS points on it or near by. Then we further adopt a matrix-factorization method^39^ to complement the speeds that cannot be directly calculated. For speed calculation, if there are more than one point on a target road, we directly calculate the speed. If there is only one point on the target road, we seek the last point (if possible) on its predecessor and the first point (if possible) on its successor. This allows us to interpolate between each pair of consecutive points to estimate the time when the vehicle enters and leaves the road. The speed is then calculated by dividing the road length by the travel time. If there is no point on the target road, we seek the points quite near the origin and destination of the road on its predecessor and successor, and similarly we infer the travel time of the target road by interpolation.

For speed complement, we view the speed calculation result as a matrix *V* with missing values, and its shape is (*N*_*r*_, *N*_*t*_) where *N*_*r*_ is the number of roads and *N*_*t*_ is the number of time slots (i.e., 24). As Eq. 5 shows, *V* is divided into two parts, *V*_*s*_ and *V*_*d*_, which account for the static component and dynamic component of the road speed respectively. *V*_*s*_ is assumed to be determined by the intrinsic road features and is further factorized into a road feature matrix *F* with shape (*N*_*r*_, *N*_*f*_) where *N*_*f*_ is the feature dimension and a feature effect matrix *E* with shape (*N*_*f*_, *N*_*t*_). We construct *F* by obtaining the road level information from OSM and the number of nearby POIs from Amap. *E* is to be learned, and each column of it is the same since the static component is independent of time. *V*_*d*_ is factorized into *R* with shape (*N*_*r*_, *N*_*t*_) and *T* with shape (*N*_*t*_, *N*_*l*_), where *N*_*l*_ is the dimension of a latent space which accounts for how road speed changes over time. *E*, *R* and *T* are learned by fitting the calculated speeds, and the missing values are complemented by the learned matrix factorization model.

5 $$V={V}_{s}+{V}_{d}=FE+{RT}^{{\rm{T}}}.$$

#### Road transition estimation

To model the prior factor (Eq. 3), we estimate the road transition probability $\Pr ({s}_{out}| {s}_{in},{r}_{e},{t}_{e})$ from an incoming road *s*_*m*_ to an outgoing *s*_*out*_ road at an intersection in the time slot of *t*_*e*_ given an camera point *r*_*e*_ as destination, based on the map-matched historical trajectories. We count the transition frequency matrix *X* at each intersection using the historical trajectory before it passes by *r*_*e*_, where the rows and columns of *X* account for the incoming roads and outgoing roads respectively. Aware that the frequency counts can be sparse given the time slot and destination, the transition probability is not estimated by directly performing normalization along the axis of outgoing roads. Instead, we adopt a uniform Dirichlet distribution as the prior to avoid too radical estimation based on few frequency counts. Therefore, with few supporting data, the transition probability is close to a uniform distribution, and with enough supporting data, the transition probability is close to the frequency tendency. We also regularize the results in each time slot with a transition probability estimated based on historical trajectories from all time slots, since it is intuitive that given the destination, driver’s routing preference is quite stable across different time of day. This regularization is helpful for time slots with fewer historical trajectories.

### Co-optimization of vehicle re-id and trajectory recovery

An iterative pipeline is implemented to jointly optimize the vehicle re-identification and trajectory recovery process. Building upon our previous work^22^, we incorporate a feedback module into the Re-ID clustering. This module leverages spatial-temporal constraints provided by the proposed probabilistic trajectory recovery model. By detecting noises and recalling missing records in the clustering result, the feedback module enhances the accuracy and feasibility of the recovered trajectory. Specifically, for noise detection, given a cluster of camera records, we find its optimal subset as non-noise records, and others as noises, using a feasibility scoring function which is based on the probabilistic spatial-temporal recovery model. Then for each detected noise, we push its dynamic feature *f*_*d*_ away from the average dynamic feature in the cluster. For missing records recalling, we try adding the detected noises from other clusters into a target cluster, and also try merging some small clusters with few records into a target cluster, if they are visually similar to the target cluster. Then we perform the noise detection as introduced above, and accept those records not detected as noise to be recalled into the target cluster. For each missing record to be recalled, we pull its dynamic feature *f*_*d*_ towards average dynamic feature in the cluster. For more details, please refer to our previous work^22^.

### Across-region trajectory recovery

After performing trajectory recovery in each city region, we further specially tackle those trajectories traveling across regions because they are truncated apart during the city division. A heuristic algorithm is designed to merge these parts. Specifically, we introduce an auxiliary region along the boundary of two adjacent regions so that it can basically cover the trajectories that travel between the two regions. Trajectories in this region are recovered by applying our recovery pipeline, and they act as links for piecing up the head and tail of a truncated trajectory across the two regions.

Formally, suppose there is a recovered trajectory ${P}_{h}^{* }$ in region *H*, which is actually the head part of an trajectory *P* truncated over region boundary. Similarly, a recovered trajectory ${P}_{t}^{* }$ in region *T* is the tail part of *P*. Given a middle part *P*_*m*_ of *P* recovered in the auxiliary region *A*, we calculate vehicle appearance feature ${f}_{a}^{m}$ and license plate feature ${f}_{p}^{m}$ as the average visual features of camera records on *P*_*m*_. Our target is to find the underlying ${P}_{h}^{* }$ in *H* and the underlying ${P}_{t}^{* }$ in *T*.

Take finding ${P}_{h}^{* }$ as example, first we construct the candidate set ${{\mathscr{P}}}_{h}$ to be those trajectories in *H* starting outside of *A* and ending inside of *A*. Then we reduce ${{\mathscr{P}}}_{h}$ by filtering out those *P*_*h*_ of which the visual similarity between ${f}_{a}^{m}$ and ${f}_{p}^{m}$ is smaller than a threshold. Next, we try matching *P*_*m*_ with each *P*_*h*_ in ${{\mathscr{P}}}_{h}$ from the spatial-temporal view. Denote the part of *P*_*h*_ and *P*_*m*_ that is within the overlapped area of *H* and *A* as ${P}_{h}^{A}$ and ${P}_{m}^{H}$ respectively. We calculate the number of trajectory points, i.e., the camera records that are in common in ${P}_{h}^{A}$ and ${P}_{m}^{H}$, which means how many times that ${P}_{h}^{A}$ and ${P}_{m}^{H}$ pass the same intersection at nearing time. Denoting the number of points in common as *a* and the number of points not in common as *b*, the matching score between *P*_*m*_ and *P*_*h*_ is calculated as:

6 $${S}_{match}=\left(\begin{array}{lc}\frac{{e}^{a}}{1+{e}^{a}}\cdot {\lambda }^{b}, & {\rm{if}}\;a > {\rm{0,}}\\ ma{x}_{p}\,{\rm{P}}{\rm{r}}(p,\Delta t| {r}_{s},{r}_{e},{t}_{e})\cdot {\lambda }^{b}, & {\rm{if}}\;a={\rm{0,}}\end{array}\right.$$

where we prefer larger *a* with a sigmoid function and punish *b* with a parameter *λ* = 0.8. And if *a* = 0 which means there is no common points between ${P}_{h}^{A}$ and ${P}_{m}^{H}$, we replace the sigmoid term with the maximum trajectory probability from the last point in ${P}_{h}^{A}$ to the first point in ${P}_{m}^{H}$ as formulated in Eq. 2. We find ${P}_{h}^{* }$ to be the *P*_*h*_ with the largest matching score between *P*_*m*_ which is at least 0.6. The matching of *P*_*m*_ and ${P}_{t}^{* }$ is similar. Through this, we link ${P}_{h}^{* }$, *P*_*m*_ and ${P}_{t}^{* }$ together to recover the whole trajectory across regions.

## Data Records

We release the city-scale vehicle trajectory data as well as the corresponding road network data at our figshare repository^40^. Both the trajectory data and the road network data are formatted as comma-separated values (CSV) files.

**Fig. 4:**
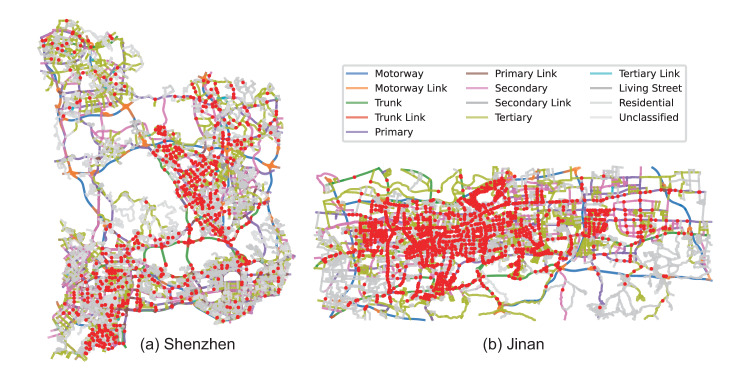
Visualization of road network data.
- **Vehicle trajectory data**. The vehicle trajectory data consists of four CSV files, one for each city and day. Three files pertain to Shenzhen city on November 4, 2020 (Wed.), April 16, 2021 (Fri.), and August 24, 2021 (Tue.), while the fourth file corresponds to Jinan city on October 17, 2022 (Mon.). Each line in the CSV file represents a trajectory, resulting in the following counts: 568,803 trajectories for Shenzhen (November 4, 2020), 1,649,085 trajectories for Shenzhen (April 16, 2021), 1,686,464 trajectories for Shenzhen (August 24, 2021), and 1,184,417 trajectories for Jinan (October 17, 2022). The data attributes and their meanings can be found in Table 3. To be specific, *VehicleID* is used to identify different vehicles. It is worth noting that the VehicleID is an enumerating index generated during the vehicle clustering process. The clustering algorithm assigns the same VehicleID to camera records within the same cluster. Therefore, the VehicleID is not associated with real-world car plates and serves as a unique identifier solely within the clustering context. The *TripID* represents the index of the trip for each vehicle. The *Points* attribute consists of trajectory points indicating road intersections and their timestamps. Additional attributes include *DepartureTime* (start time), *Duration* (time duration), and *Length* (travel length) of each trajectory.

- **Road network data**. The road network data is provided separately for Shenzhen city and Jinan city, with each city having two CSV files: one for graph nodes and another for graph edges. Table 2 presents the attributes and meanings of the data contained in the CSV files, providing a comprehensive understanding of the road network data for both cities.
  - **Node CSV file**. Each line contains information about a road intersection, including a unique NodeID, the Longitude and Latitude coordinates in the WGS84 format, and a binary indicator (HasCamera) for the presence of cameras at the intersection. Specifically, in Shenzhen, there are 11,933 intersections, while in Jinan, there are 8,908 intersections.
  - **Edge CSV file**. Each line represents a specific road segment and provides information such as the origin and destination NodeID, road class obtained from OpenStreetMap (OSM), geometry (coordinates in WGS84 format) defining the shape of the segment, and its length in meters. In Shenzhen, there are 27,410 road segments, and in Jinan, there are 23,312 road segments.
  The road network data is visualized in Fig. 4. In the visualization, the road intersections with a “HasCamera” attribute of “1” are marked as red spots. These intersections are equipped with cameras for monitoring and surveillance purposes. The roads are plotted based on the “Geometry” attribute, which provides the coordinates of each point along the road linestring. The color scheme is used to distinguish different road classes based on the “Class” attribute. Each road class is assigned a specific color, allowing for easy identification and differentiation of road types.

**Table 2:** Road network data attributes.

		Meaning	Example	Notes
Node	NodeID	Node Identifier	0	An index code
	Longitude	Longitude of the intersection	114.02342	In World Geodetic System-1984 (WGS84)
	Latitude	Latitude of the intersection	22.62788	In World Geodetic System-1984 (WGS84)
	HasCamera	Whether there is any camera deployed at this intersection	0	“0” if no cameras, “1” otherwise
Edge	Origin	NodeID of the edge’s origin	1	
	Destination	NodeID of the edges’s destination	2	
	Class	Road class information from OSM	primary	Refer to OSM wiki for notes: https://wiki.openstreetmap.org/wiki/Map_features#Roads
	Geometry	Coordinates of the road linestring. Points are divided by “_”.	114.0221-22.6414_114.0258-22.6404	In World Geodetic System-1984 (WGS84)
	Length	Length of the road linestring	400	In meters

**Table 3:** Trajectory data attributes.

	Meaning	Example	Notes
VehicleID	Vehicle Identifier	0	An enumerating index generated during the vehicle clustering rather than car plate.
TripID	Index of trip of the vehicle	0	A vehicle may have multiple trips in a day, so “VehicleID” is not enough to uniquely identify a trajectory.
Points	A series of trajectory points. Each point is “NodeID-Time”. Points are divided by “_”.	1-36000_3-36300_6-36900	“NodeID” is a intersection corresponding to road network. Exactly one road segment existing between two adjacent nodes is guaranteed. “Time” is represented by seconds starting from the beginning of the day.
DepartureTime	Departure time of the trajecotry	36000	“Time” is represented by seconds starting from the beginning of the day.
Duration	Time duration of the trajectory	900	In seconds
Length	Travel length of the trajectory	5000	In meters

## Technical Validation

In this section, we aim to validate the quality of the trajectory data recovered from videos and demonstrate that our proposed dataset contains almost full-amount of vehicle trajectories. We begin by evaluating the performance of our trajectory recovery method through an assessment of the resulting trajectories. Then, we examine the robustness of our method and discuss how traffic camera density can impact the quality of trajectory data by comparing the performance in different city regions with varied traffic camera density. To gain insights into the quality of the trajectories, we conduct an individual-level analysis by studying specific cases. This analysis allows us to examine the trajectories at a granular level and assess their accuracy and reliability. Furthermore, we analyze aggregated-level features such as road speed and flow. This broader analysis provides a comprehensive understanding of the overall characteristics of the trajectories and helps evaluate their usefulness in capturing traffic patterns and vehicle movements. By conducting these evaluations, we ensure the reliability and completeness of our dataset.

### The performance of vehicle trajectory recovery

We collect vehicle trajectory ground truth on August 24, 2021 in Shenzhen city, which contains the GPS trajectory of 423 vehicles and 15,081 corresponding traffic camera records with identity labels. We evaluate the performance of our work on both the vehicle Re-ID task and the trajectory recovery task, since the prerequisite Re-ID task strongly affects the performance of the final trajectory recovery task. For the vehicle Re-ID task, we measure the precision, recall, F1-score and expansion of clustering results as our previous work proposes^22^. For the trajectory recovery task, we use metrics including Longest Common SubSequence (LCSS), Edit Distance on Real sequence (EDR) and Spatial-Temporal Linear Combine distance (STLC)^41^. We compare the performance of our method with baselines including BNN^42^, VeTrac^26^ and MMVC^21^, and for implementation details please refer to our previous work^22^. As Table 4 shows, our method achieves satisfactory overall performance and consistently outperforms all the baselines on both tasks across various metrics.

**Table 4:** Overall performance of our vehicle trajectory recovery approach and the baselines.

Method	Vehicle Re-ID				Trajectory Recovery		
	Precision	Recall	F1-score	Expansion	LCSS	EDR	STLC
BNN	0.6311	0.7002	0.6639	3.6427	0.8315	58.6667	0.4940
VeTrac	0.7022	0.8236	0.7581	2.3813	0.7242	30.0413	0.6393
MMVC	0.8448	0.8690	0.8567	2.1940	0.6932	21.0645	0.6963
Ours	**0.8545**	**0.8721**	**0.8632**	**2.1632**	**0.6778**	**17.0399**	**0.7160**

### The impact of traffic camera density on the quality of vehicle trajectories

To further examine the robustness of the method and the consistency of the trajectory quality across heterogeneous scenarios, we study five distinct city regions in Shenzhen. These regions exhibit varying traffic camera densities, and we compare their performance using the same metrics as mentioned earlier. These regions correspond to the city division results previously introduced. The density is defined as the proportion of road intersections covered by traffic cameras, specifically denoting the fraction of road intersections equipped with any traffic camera. During the statistical analysis, we filter out intersections located between “residential”, “living street” and “unclassified” roads. Instead, we only concentrate on the traffic camera coverage rate among other intersections, in order to control over the extent to which residential details are included in the OSM road network across different city regions. This is important since traffic cameras are not expected to be deployed at residential intersections.

We show the results in Table 5 and draw three key observations. First, the city regions with top three traffic camera densities have similarly satisfactory performance, whereas those with the lowest two traffic camera densities show relatively poorer performance. As expected, higher traffic camera density correlates with better trajectory quality, measured from both the vehicle Re-ID task and the trajectory recovery task. Second, with increasing traffic camera density, the quality of vehicle Re-ID initially improves before slightly declining, with the third city region generally demonstrating the best performance. This pattern is both intriguing and reasonable. When camera density is too low, the performance of trajectory recovery is inherently poor. As a result, our co-optimization module can hardly leverage weak spatial-temporal constraints to guide the Re-ID clustering. Conversely, when camera density is excessively high, the clustering task becomes more challenging due to the increased number of camera records to be clustered, potentially leading to a drop in performance. Third, the trajectory recovery quality follows a similar trend of initially rising and then slightly declining with increasing traffic camera density. In this case, the second city region generally displays the best performance. This is reasonable because the difficulty of the trajectory recovery task is primarily influenced by the coverage rate of traffic cameras. Additionally, the quality of the upstream task, namely vehicle Re-ID, also impacts the final outcome. Moreover, the observation that the second region excels in trajectory recovery while the third region excels in vehicle Re-ID aligns with the insight that trajectory recovery benefits from higher camera density, whereas vehicle Re-ID is more favorable with a lower camera density to ensure manageable clustering tasks.

**Table 5:** Comparison of performance under different traffic camera density.

Camera Density	Vehicle Re-ID				Trajectory Recovery		
	Precision	Recall	F1-score	Expansion	LCSS	EDR	STLC
0.2573	0.8767	0.8646	0.8706	2.6502	0.6224	15.8000	0.7548
0.1549	0.8888	0.8867	0.8877	1.6949	**0.5449**	**15.1100**	**0.7995**
0.1022	**0.8924**	**0.9107**	**0.9014**	1.9829	0.6184	15.7857	0.7760
0.0819	0.8026	0.8558	0.8283	**1.2654**	0.7310	19.9091	0.6828
0.0638	0.8411	0.8603	0.8506	2.5812	0.7570	18.0656	0.6495

To sum up, our method demonstrates robust performance across diverse scenarios, with performance discrepancies attributed to varying traffic camera densities, supported by insightful explanations. This preliminary study shows that in Shenzhen, the quality of trajectory data remains satisfactory when traffic camera density exceeds 10%. However, it is noteworthy that the data quality may experience a decline (but remain comparable to the city-scale overall performance of the best baseline as shown in Table 4) when camera density is small.

### A case study to verify the quality of vehicle trajectories

**Fig. 5:**
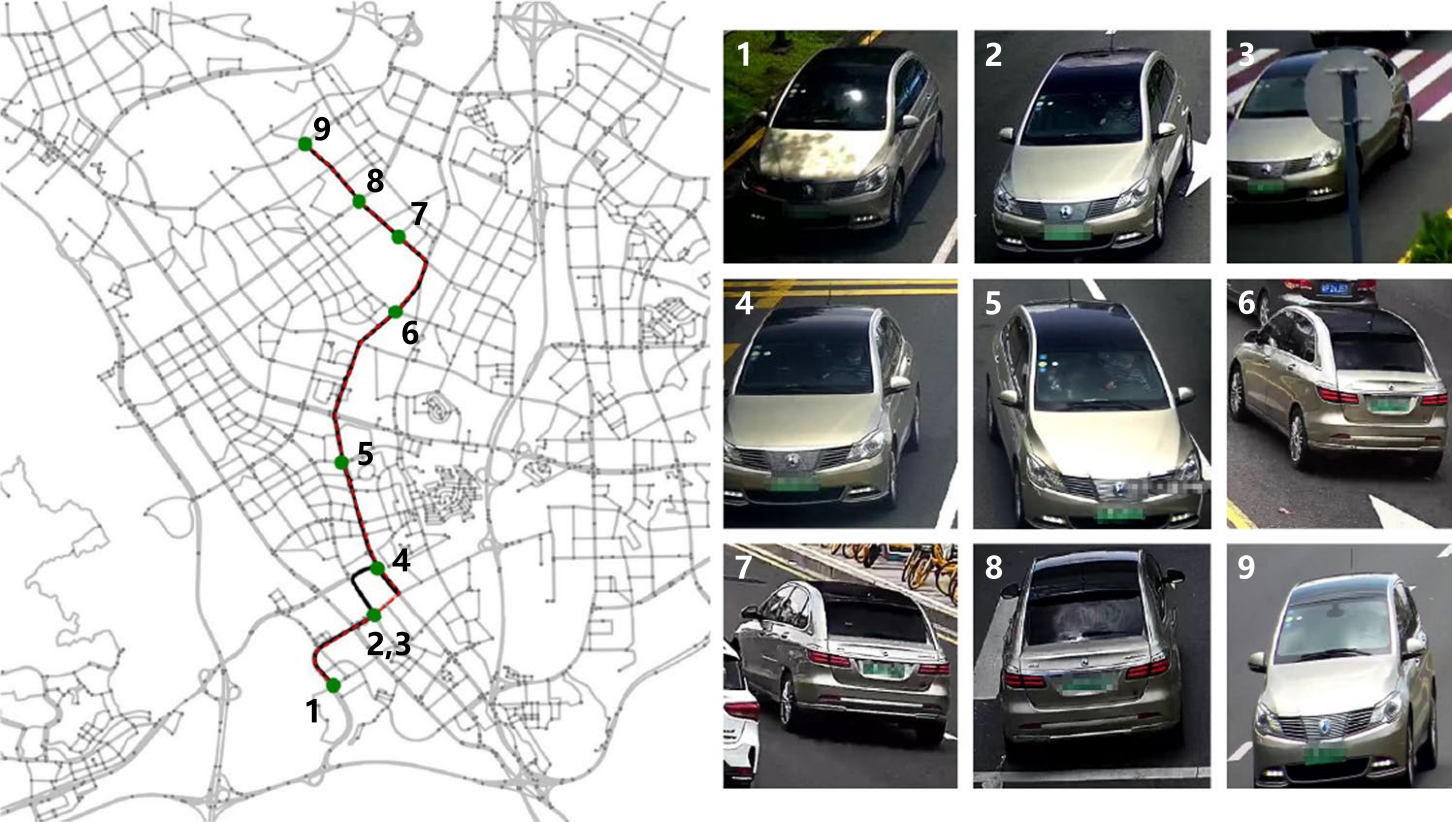
A recovered vehicle trajectory and its related image records in Shenzhen.

**Fig. 6:**
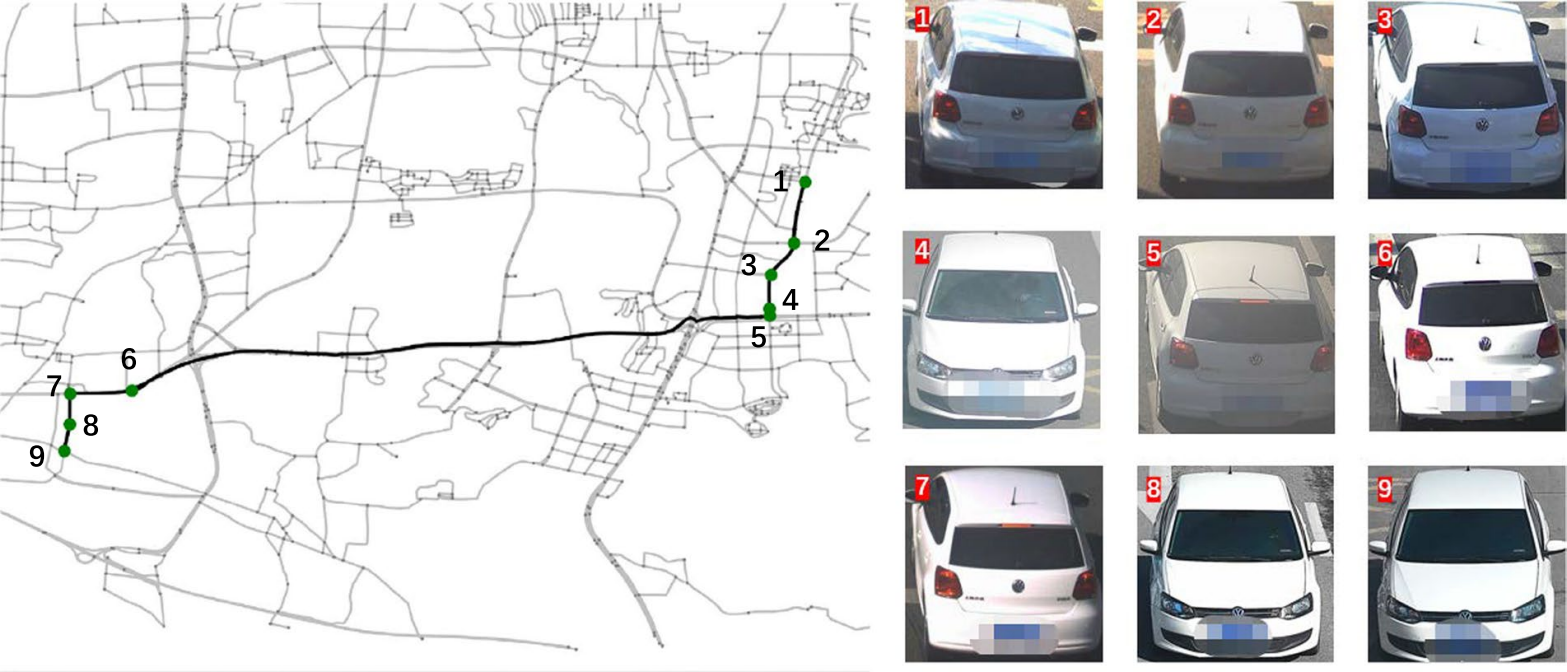
A recovered vehicle trajectory and its related image records in Jinan.

To assess the quality of the recovered trajectories, we provide a specific example from Shenzhen along with the corresponding traffic camera records and the ground truth trajectory. In Fig. 5, the red dashed line represents the ground truth trajectory, which spans a distance of over 10 km. The black solid line indicates the recovered trajectory, which closely aligns with the ground truth. Furthermore, all nine traffic camera records (depicted as green nodes) along the trajectory path are accurately matched, despite challenges such as a large number of camera records, varying shooting angles, and unfavorable lighting conditions (e.g., record 1). This case demonstrates the effectiveness of our vehicle Re-ID and trajectory recovery framework, affirming the satisfactory quality of our vehicle trajectory data based on traffic camera videos. In the case of Jinan, since there were no real trajectories available in the dataset, we only presented the recovered trajectories along with the corresponding images of vehicles captured by cameras. These images are displayed in Fig. 6. By examining the vehicle images, it is evident that they belong to the same vehicle, demonstrating the accuracy of our trajectory recovery algorithm.

### Statistical analysis of vehicle trajectories

To gain valuable insights into the statistical characteristics of trajectory data within the city, we conducted visualizations focusing on two perspectives: the static view and the dynamic view.

#### Static view

**Fig. 7:**
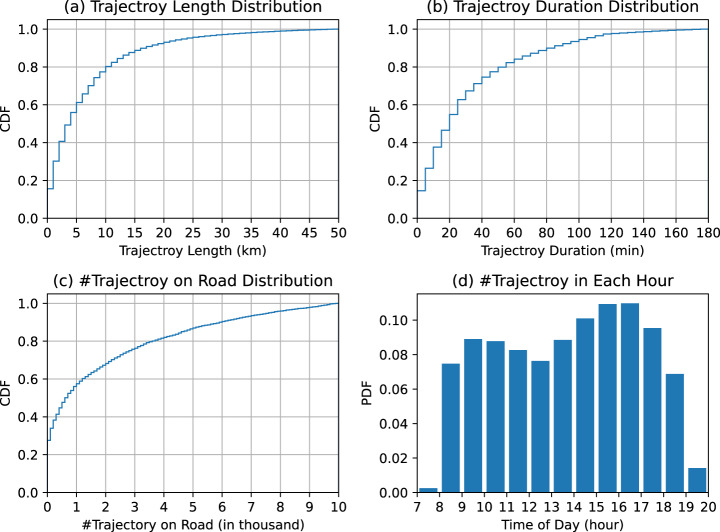
Visualization of statistical characteristics of the recovered trajectories in Shenzhen.

**Fig. 8:**
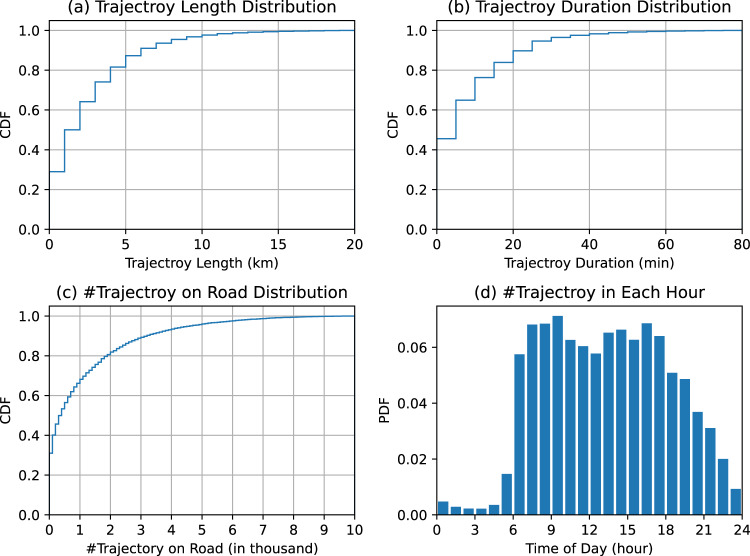
Visualization of statistical characteristics of the recovered trajectories in Jinan.

We conducted a spatial-temporal analysis of trajectory data in both Shenzhen and Jinan. Figure 7 shows the statistics for Shenzhen, including the cumulative distribution function (CDF) figures for trajectory length, duration, and quantity on roads, which exhibit long-tailed characteristics. Figure 7d displays the number of trajectories departing in each hour, indicating a concentration of trajectories between 8 a.m. and 7 p.m., with clear morning and evening peaks. However, trajectories from 6 p.m. to 7 p.m. may not appear as a significant peak due to limited data availability after 7 p.m. Figure 8 presents the statistics for Jinan, where the distributions of trajectory length, duration, and quantity on roads show a similar long-tailed pattern as in Shenzhen. Figure 8d illustrates the number of trajectories departing in each hour in Jinan, revealing two peaks during rush hours (8:00–9:00 am and 4:00–5:00 pm), a trough during noon, and minimal vehicle activity at night, aligning with the expected traffic patterns.

#### Dynamic view

**Fig. 9:**
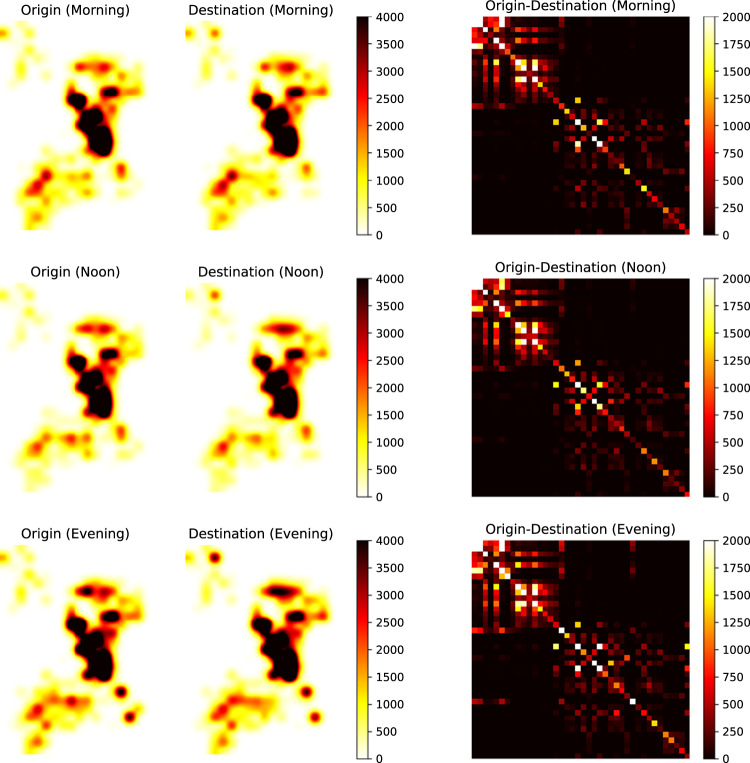
Flow distribution of different OD pairs at different time periods in Shenzhen.

**Fig. 10:**
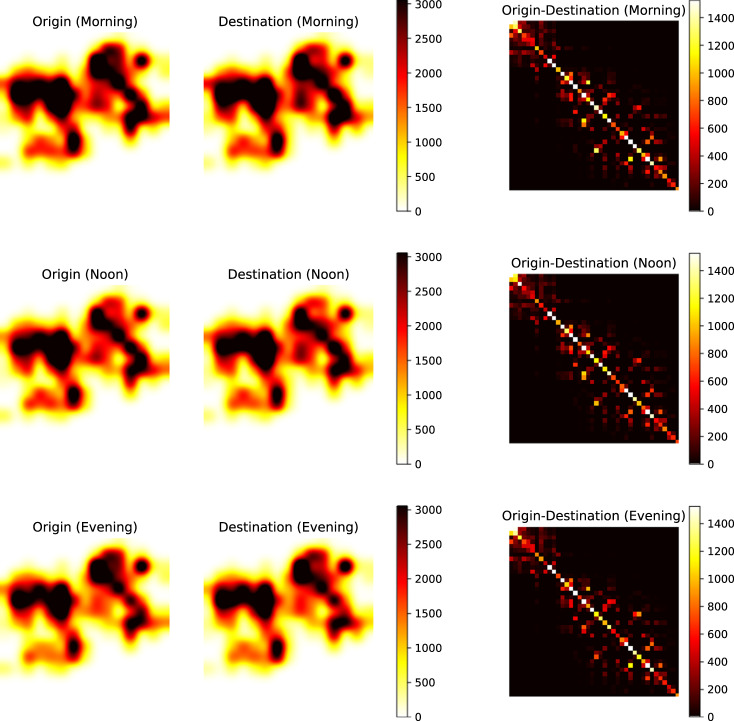
Flow distribution of different OD pairs at different time periods in Jinan.

We present the heat maps of origin distribution (O), destination distribution (D), and origin-destination (OD) flow for vehicle trajectories in Shenzhen and Jinan (Figs. 9, 10). The columns represent O, D, and OD, while the rows correspond to morning, noon, and evening periods. In Shenzhen, we analyzed trajectories during three 2-hour windows (9:00–11:00, 12:00–14:00, and 16:00–18:00). The top 40 grids with the highest O and D flows, accounting for 74% of the total flows, were selected. The resulting O and D figures exhibit consistent traffic patterns, aligning with the distribution of traffic cameras and showing increased activity and diverse mobility patterns during peak hours. Similarly, in Jinan, we analyzed trajectories during three 2-hour windows (9:00–11:00, 12:00–14:00, and 16:00–18:00). The top 40 grids with the highest O and D flows, accounting for 67% of the total flows, were selected. The O and D figures demonstrate that more hot spots appear during peak hours, indicating increased activity and similar traffic patterns to Shenzhen.

### Feature analysis of aggregated vehicle trajectories

In this subsection, we aggregate the vehicle trajectories to further validate the data quality and demonstrate that our dataset encompasses almost all vehicles in the city during the specified time range.

**Fig. 11:**
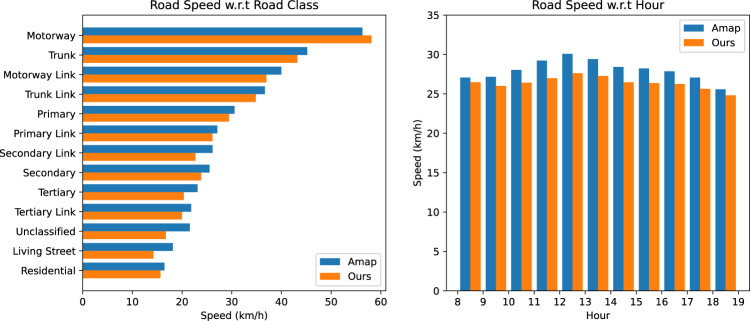
Comparison of road speeds between our released data and Amap data for different types of roads in Shenzhen.

**Fig. 12:**
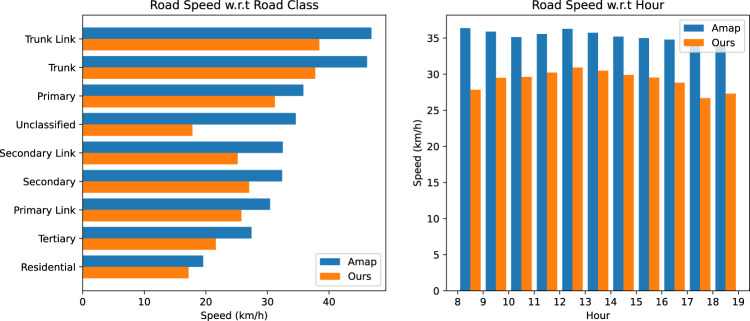
Comparison of road speeds between our released data and Amap data for different types of roads in Jinan.

We first validate data quality by comparing our road speeds with Amap’s. Road speeds are estimated by calculating average speeds for each road segment using trajectory points at both ends. We filter out roads with less than 1,000 speed values and remove outliers based on Median Absolute Deviation^43^. To visualize the comparison results, we aggregate the speeds w.r.t road class and hour respectively, as shown in Fig. 11 (Shenzhen) and Fig. 12 (Jinan). Both Amap and our speeds show expected differences among different road types, with our speeds slightly lower. Our speed calculation includes waiting time at traffic lights and midway stops, while Amap’s data is based on instant GPS speeds. When comparing speeds by hour, a noon-time peak is observed in both datasets, with our speeds generally slightly smaller than Amap’s. We also report Mean Absolute Error (MAE) and Root Mean Square Error (RMSE) values for each road and hour. In Shenzhen, MAE and RMSE values are 7.13 km/h and 9.39 km/h, respectively. In Jinan, MAE and RMSE values are 7.66 km/h and 9.82 km/h, respectively. It’s important to note that the Amap speed data we compared with was averaged from historical days, not collected on the exact same day as our dataset. The reported MAE and RMSE values are reasonable considering daily variations in road speeds.

**Fig. 13:**
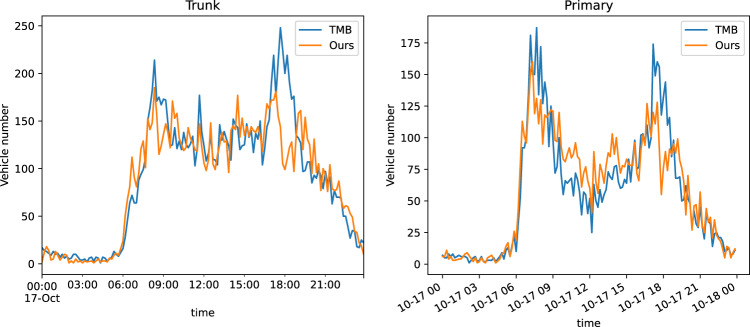
Comparison of vehicle numbers on two types of roads (our released data V.S. TMB data).

In terms of data quantity, our trajectory dataset represents a city-scale coverage, including nearly the full amount of vehicles in the city region during the specified time range. In Shenzhen, on April 16, 2021, there were 1,649,085 trajectories from 1,121,683 vehicles, and on August 24, 2021, there were 1,686,464 trajectories from 1,103,997 vehicles. The recovered number of vehicles accounts for approximately 44% of the total daily vehicle count reported by the local government, considering factors such as limited camera coverage and collection time. In Jinan, comparing our released data with the local traffic management bureau (TMB) data, we found similar temporal patterns and a small discrepancy in vehicle counts shown in Fig. 13, with an average MAE of approximately 18 for trunk roads and 14 for primary roads. There are 1,184,417 recovered trajectories with 963,125 vehicles in total. This closely aligns with the average number of weekday vehicles reported by TMB, which is around 0.89 million.

## Usage Notes

All datasets open in this paper are in file form, and users can access them in their entirety without any further permission. For your convenient usage, we recommend some Python packages for processing and analyzing the released datasets conveniently. First, Pandas^44^ can be used to read and process the CSV files. Then, we suggest using NetworkX^45^ to construct a topology graph of the road network, which can synthesize the files for both nodes and edges. With the road network graph, users can easily convert the trajectory, which is described by the “NodeID” of road intersections, to a GPS coordinates based trajectory. Next, we recommend Folium^46^ for visualizations of trajectories or road network with background map tiles. For further geometric computations or operations on our datasets, e.g., interpolating the intersection-level trajectory along the road geometry to obtain denser trajectory point, we suggest Shapely^47^ with rich API for geometry objects and computations. Besides, if you have any problems with coordinate reference system conversion, e.g., combining our data with other data sources with possible different coordinate systems, we recommend Pyproj^48^. An example to read, convert and visualize our dataset is also provided in our GitHub repository^49^.

Finally, we discuss the limitations of the dataset and the potential restriction to its applications, helping researchers decide whether it is suitable for their studies. First, because the dataset involves only four days of trajectories, it cannot support studies focused on long-term mobility patterns, such as city growth involving evolving mobility patterns^50^. For analytical research that tries to draw general conclusions such as the yearly average distribution of vehicle exhaust^51^, statistical road flow and speed data is more suitable. However, our dataset contains short-term and fine-grained traffic dynamics, and offers comprehensive insights into specific days. It proves useful for traffic congestion modeling^27^, where short-term dynamics are crucial, or for cross-sectional studies trading generalization performance for more precise and detailed calculations^28^. For methodology oriented studies which mainly use trajectory data to develop and validate the methods rather than make explicit practical management decisions, the dataset remains suitable as long as the method itself can operate spanning only a few days^31,32^. Second, trajectories recorded by traffic cameras cannot strictly represent a vehicle’s entire journey due to potential loss of data concerning the “first mile” before the first camera and the “last mile” after the last camera. Therefore, for studies requiring precise origin and destination information of trajectories, the dataset may be not suitable.

## Data Availability

The codes for how we generate the trajectory dataset based on visual embedded traffic camera records, evaluate the vehicle Re-ID and trajectory recovery metrics, and report statistical characteristics are available in our GitHub repository^49^. There are also tips for installing Python requirements.

## References

[1] Kong X (2018). Lotad: Long-term traffic anomaly detection based on crowd sourced bus trajectory data. World Wide Web.

[2] Meng, C., Yi, X., Su, L., Gao, J. & Zheng, Y. City-wide traffic volume inference with loop detector data and taxi trajectories. In *Proceedings of the 25th ACM SIGSPATIAL International Conference on Advances in Geographic Information Systems*, 1–10 (2017).

[3] Yin X (2022). Deep learning on traffic prediction: Methods, analysis, and future directions. IEEE Transactions on Intelligent Transportation Systems.

[4] Li F (2023). Dynamic graph convolutional recurrent network for traffic prediction: Benchmark and solution. ACM Transactions on Knowledge Discovery from Data.

[5] Jin, G., Li, F., Zhang, J., Wang, M. & Huang, J. Automated dilated spatio-temporal synchronous graph modeling for traffic prediction. *IEEE Transactions on Intelligent Transportation Systems* (2022).

[6] Zheng, G. *et al*. Learning phase competition for traffic signal control. In *Proceedings of the 28th ACM international conference on information and knowledge management*, 1963–1972 (2019).

[7] Ma W, Wan L, Yu C, Zou L, Zheng J (2020). Multi-objective optimization of traffic signals based on vehicle trajectory data at isolated intersections. Transportation research part C: emerging technologies.

[8] Wang J, Peeta S, He X (2019). Multiclass traffic assignment model for mixed traffic flow of human-driven vehicles and connected and autonomous vehicles. Transportation Research Part B: Methodological.

[9] New York Taxi and Limousine Commission. *New york city taxi trip data.* https://www.nyc.gov/site/tlc/about/tlc-trip-record-data.page (2023).

[10] Zhang, J., Zheng, Y. & Qi, D. Deep spatio-temporal residual networks for citywide crowd flows prediction. In *Proceedings of the AAAI conference on artificial intelligence*, vol. 31 (2017).

[11] Zhao L (2020). T-gcn: A temporal graph convolutional network for traffic prediction. IEEE Transactions on Intelligent Transportation Systems.

[12] Yuan, J. *et al*. T-drive: Driving directions based on taxi trajectories. In *Proceedings of 18th ACM SIGSPATIAL Conference on Advances in Geographical Information Systems* (ACM SIGSPATIAL GIS 2010, 2010).

[13] Liao, B. *et al*. Deep sequence learning with auxiliary information for traffic prediction. In *Proceedings of the 24th ACM SIGKDD International Conference on Knowledge Discovery and Data Mining*, 537–546 (ACM, 2018).

[14] The DiDi GAIA Initiative. *Didi chuxing gaia open dataset.* https://gaia.didichuxing.com/ (2019).

[15] Department of Transportation Federal Highway Administration. Next generation simulation (ngsim) vehicle trajectories and supporting data, 10.21949/1504477 (2016).

[16] He Y, Cao B, Chan C-Y (2022). Wut-ngsim: A high-precision and trustworthy vehicle trajectory dataset.

[17] Zhao, D. & Li, X. Real-world trajectory extraction from aerial videos-a comprehensive and effective solution. In *2019 IEEE Intelligent Transportation Systems Conference (ITSC)*, 2854–2859 (IEEE, 2019).

[18] Shi X (2021). Video-based trajectory extraction with deep learning for high-granularity highway simulation (high-sim). Communications in transportation research.

[19] Zheng, O., Abdel-Aty, M., Yue, L., Abdelraouf, A., Wang, Z., & Mahmoud, N. (2023). CitySim: A Drone-Based Vehicle Trajectory Dataset for Safety-Oriented Research and Digital Twins. *Transportation Research Record*, 0(0), 10.1177/03611981231185768

[20] Krajewski, R., Bock, J., Kloeker, L. & Eckstein, L. The highd dataset: A drone dataset of naturalistic vehicle trajectories on german highways for validation of highly automated driving systems. In *2018 21st International Conference on Intelligent Transportation Systems (ITSC)*, 2118–2125 (IEEE, 2018).

[21] Lin, Z. *et al*. Vehicle trajectory recovery on road network based on traffic camera video data. In *Proceedings of the 29th International Conference on Advances in Geographic Information Systems*, 389–398 (2021).

[22] Yu, F. *et al*. Spatio-temporal vehicle trajectory recovery on road network based on traffic camera video data. In *Proceedings of the 28th ACM SIGKDD Conference on Knowledge Discovery and Data Mining*, 4413–4421 (2022).

[23] Wang Y (2023). City-scale holographic traffic flow data based on vehicular trajectory resampling. Scientific Data.

[24] Li G (2023). City-scale synthetic individual-level vehicle trip data. Scientific Data.

[25] Shen, Y., Xiao, T., Li, H., Yi, S. & Wang, X. Learning deep neural networks for vehicle re-id with visual-spatio-temporal path proposals. In *Proceedings of the IEEE international conference on computer vision*, 1900–1909 (2017).

[26] Tong, P., Li, M., Li, M., Huang, J. & Hua, X. Large-scale vehicle trajectory reconstruction with camera sensing network. In *Proceedings of the 27th Annual International Conference on Mobile Computing and Networking*, 188–200 (2021).

[27] Bellocchi L, Geroliminis N (2020). Unraveling reaction-diffusion-like dynamics in urban congestion propagation: Insights from a large-scale road network. Scientific reports.

[28] Böhm M, Nanni M, Pappalardo L (2022). Gross polluters and vehicle emissions reduction. Nature Sustainability.

[29] Hamedmoghadam H, Jalili M, Vu HL, Stone L (2021). Percolation of heterogeneous flows uncovers the bottlenecks of infrastructure networks. Nature communications.

[30] Wu, G. *et al*. Human-centric urban transit evaluation and planning. In *2018 IEEE International Conference on Data Mining (ICDM)*, 547–556 (IEEE, 2018).

[31] Kavianipour M (2021). Electric vehicle fast charging infrastructure planning in urban networks considering daily travel and charging behavior. Transportation Research Part D: Transport and Environment.

[32] Kong W, Luo Y, Feng G, Li K, Peng H (2019). Optimal location planning method of fast charging station for electric vehicles considering operators, drivers, vehicles, traffic flow and power grid. Energy.

[33] OpenStreetMap contributors. Planet dump retrieved from https://planet.osm.org. https://www.openstreetmap.org (2017).

[34] Boeing G (2017). Osmnx: New methods for acquiring, constructing, analyzing, and visualizing complex street networks. Computers, Environment and Urban Systems.

[35] Wikipedia contributors. Address geocoding–Wikipedia, the free encyclopedia. https://en.wikipedia.org/wiki/Address_geocoding (2023).

[36] Jing, W. Size constrained clustering. https://github.com/jingw2/size_constrained_clustering (2020).

[37] Johnson J, Douze M, Jégou H (2019). Billion-scale similarity search with GPUs. IEEE Transactions on Big Data.

[38] Yang C, Gidofalvi G (2018). Fast map matching, an algorithm integrating hidden markov model with precomputation. International Journal of Geographical Information Science.

[39] Wu, H. *et al*. Probabilistic robust route recovery with spatio-temporal dynamics. In *Proceedings of the 22nd ACM SIGKDD International Conference on Knowledge Discovery and Data Mining*, 1915–1924 (2016).

[40] Yu F (2023). figshare.

[41] Su H, Liu S, Zheng B, Zhou X, Zheng K (2020). A survey of trajectory distance measures and performance evaluation. The VLDB Journal.

[42] Luo H (2019). A strong baseline and batch normalization neck for deep person re-identification. IEEE Transactions on Multimedia.

[43] Wikipedia contributors. *Median absolute deviation–Wikipedia, the free encyclopedia.* https://en.wikipedia.org/wiki/Median_absolute_deviation (2023).

[44] The Pandas Development Team (2020). pandas-dev/pandas: Pandas.

[45] Hagberg, A. A., Schult, D. A. & Swart, P. J. Exploring network structure, dynamics, and function using networkx. In Varoquaux, G., Vaught, T. & Millman, J. (eds.) *Proceedings of the 7th Python in Science Conference*, 11–15 (Pasadena, CA USA, 2008).

[46] The Folium Development Team. Folium: Python data, leaflet.js maps. https://python-visualization.github.io/folium/ (2020).

[47] Gillies, S. *et al*. *Shapely: manipulation and analysis of geometric objects.* https://github.com/Toblerity/Shapely (2007).

[48] The Pyproj Development Team. *Pyproj: Python interface to proj (cartographic projections and coordinate transformations library).* https://github.com/pyproj4/pyproj (2020).

[49] Yu, F. *et al*. The offcial implementation of the paper “city-scale vehicle trajectory data from traffic camera videos”. https://github.com/tsinghua-fib-lab/City-Camera-Trajectory-Data (2023). 10.1038/s41597-023-02589-y PMC10582153 37848455

[50] Xu F, Li Y, Jin D, Lu J, Song C (2021). Emergence of urban growth patterns from human mobility behavior. Nature Computational Science.

[51] Chen S (2021). Moves-beijing-based high spatial and temporal resolution ammonia emissions from road traffic in beijing. Atmospheric Environment.

